# Does encoding matter? A novel view on the quantitative genetic trait prediction problem

**DOI:** 10.1186/s12859-016-1127-1

**Published:** 2016-07-19

**Authors:** Dan He, Laxmi Parida

**Affiliations:** IBM T.J. Watson Research, Yorktown Heights, NY USA

**Keywords:** Quantitative genetic trait prediction, Encoding, Epistasis, Ridge regression

## Abstract

**Background:**

Given a set of biallelic molecular markers, such as SNPs, with genotype values encoded numerically on a collection of plant, animal or human samples, the goal of genetic trait prediction is to predict the quantitative trait values by simultaneously modeling all marker effects. Genetic trait prediction is usually represented as linear regression models which require quantitative encodings for the genotypes. There are lots of work on the prediction algorithms, but none of the existing work investigated the effects of the encodings on the genetic trait prediction problem.

**Methods:**

In this work, we view the genetic trait prediction problem from a novel angle: a multiple regression on categorical data problem, which requires encoding the categorical data into numerical data. We further proposed two novel encoding methods and we show that they are able to generate numerical features with higher predictive power.

**Results and Discussion:**

Our experiments show that our methods are superior to the other encoding methods for both single marker model and epistasis model. We showed that the quantitative genetic trait prediction problem heavily depends on the encoding of genotypes, for both single marker model and epistasis model.

**Conclusions:**

We conducted a detailed analysis on the performance of the hybrid encodings. To our knowledge, this is the first work that discusses the effects of encodings for genetic trait prediction problem.

## Background

Whole genome prediction of complex phenotypic traits using high-density genotyping arrays has attracted a lot of attention, as it is relevant to the fields of plant and animal breeding and genetic epidemiology [1–8]. Given a set of biallelic molecular markers, such as SNPs, with genotype values typically encoded as {0,1,2} on a collection of plant, animal or human samples, the goal is to predict the quantitative trait values by simultaneously modeling all marker effects.

More specifically, the genetic trait prediction problem is defined as follows. Given *n* training samples, each with *m*≫*n* genotype values (we use “feature”, “marker”, “genotype”, “SNP” interchangeably) and a trait value, and a set of *n*^′^ test samples each with the same set of genotype values but without trait value, the task is to train a predictive model from the training samples to predict the trait value, or phenotype of each test sample based on their genotype values. Let *Y* be the trait value of the training samples. The problem is usually represented as the following linear regression model:

1 $$ Y = \beta_{0} + \sum_{i=1}^{m} \beta_{i} X_{i} + e_{l}   $$

where *X*_*i*_ is the *i*-th genotype value, *m* is the total number of genotypes and *β*_*i*_ is the regression coefficient for the *i*-th genotype, *e*_*l*_ is the error term. We call this model *single marker model*.

The above model assumes that only the single markers, or main effects, play a role for the prediction. However, it is known that the interactions of the markers may also contribute to the genetic traits under certain conditions, which is known as *Epistasis* [15]. The pairwise epistasis between two markers *i* and *j* is often modeled as the product of the two genotype values. Therefore, with the traditional representation, the linear regression model with pairwise epistasis effects is modified as the following:

2 $$ Y= \beta_{0} + \sum_{i=1}^{m} \beta_{i} X_{i} + \sum_{i,j}^{m} \alpha_{i,j} X_{i} X_{j} + e_{l}   $$

where *X*_*i*_*X*_*j*_ is the product of the genotype values of the *i*-th and *j*-th genotype and it denotes the interaction of the two genotypes, *α*_*i*,*j*_ represents the coefficient for the interaction. Thus in this epistasis model, the epistasis effects are considered as augmented genotypes besides the original genotype matrix *X*. We call this model *epistasis model*. As multiplication is one of the most popular epistasis models, in this work, we consider only the multiplication model for epistasis.

Genotypes for a marker can be either homozygous or heterozygous. For Genome Wide Association Study (GWAS), we only need to identify the association between a marker and the case/control trait. Therefore, we care more about whether genotypes are homozygous or heterozygous, the specific alleles and their frequencies. The genotypes don’t necessarily need to be quantitative. They are usually represented as a pair of alleles, for example “AA" and “TT" for homozygous genotypes, “AT" for heterozygous genotype.

On the other hand, for genetic trait prediction problem, in Eq. 1, the genetic trait values *Y* are quantitative. Thus the genotypes *X*_*i*_ needs to be quantitative as well. Researchers generally assign three distinct encodings to the three possible genotype values. A few common sets of encodings for genotypes are {0,1,2}, where 0 and 2 are for homozygous genotypes and 1 is for heterozygous genotype, and {−1,0,1}, where -1 and 1 are for homozygous genotypes and 0 is for heterozygous genotype.

There have been lots of work on predicting genetic trait values from genotype data, such as rrBLUP (Ridge regression BLUP) [1], Elastic-Net, Lasso, Ridge Regression [10, 11], Bayes A, Bayes B [1], Bayes *C*_*π*_ [12], and Bayesian Lasso [13, 14], as well as other machine learning methods. However, all previous work consider genetic trait prediction problem as a regression problem on a numerical data set. We, on the contrary, look at the problem from a totally different angle: we consider the problem as a problem of *multiple regression on categorical data*, namely a regression on multiple categorical features. The genotype of each marker has three possible categories: homozygous with major allele, homozygous with minor allele and heterozygous. In order to conduct regression on categorical data, we need to first encode the categorical data. Many encoding methods have been proposed for categorical data, including dummy encoding, ordinal encoding, target-based encoding etc. The traditional coding of {0,1,2} is indeed the ordinal coding, which assumes that the categories follow certain order. In this problem setting, the three categories can be considered as following the order of the number of major or minor alleles.

In this work, we first review the existing encoding mechanisms and we show that only ordinal encoding and target-based encoding are appropriate for the genetic trait prediction problem. The ordinal encoding encodes the three categories into three unique numerical values, such as {0,1,2} or {-1,0,1}. The advantage of the ordinal encoding is that the order of the categories are maintained by the encoding. The target-based encoding encodes the three categories as follows: For each category *g* of each marker *i*, we identify the set of trait values for the samples whose category is *g* at marker *i*. Then we take the average of this set of traits and assign the genotype with this average value. The advantage of the target-based encoding is that each marker, or feature, can be encoded differently according to the data. We also observe that the ordinal encoding method always encodes different markers with the same set of numerical values. On the contrary, the target-based encoding methods can not maintain the order of the categories. To combine the advantages of both mechanisms and to address their drawbacks at the same time, we further developed two hybrid encoding methods. The hybrid methods conduct target-based encoding for the two homozygous categories first, then encode the heterozygous category either as the mean of the trait for all samples, or the mean of the two homozygous categories. Thus they allow the flexibility of the encodings, where different markers can be encoded differently. We showed that the encoded value for the heterozygous category is always bounded by the two values of the homozygous categories. Therefore the hybrid encoding methods maintain the order of the categories. We also extended these hybrid encoding methods to epistasis model. We showed that our hybrid encoding methods are superior to both ordinal and target-based encodings for both single marker model as well as epistasis model. Due to space limit, we did not include the experimental results for the epistasis model, which will be included in the extended version of the work.

## Preliminaries

Given the traditional encoding of genotypes as {0,1,2}, lots of techniques have been applied to the genetic trait prediction problem defined in Eq. 1. Consider the typical situation for linear regression, where we have the training set $\mathbf {y} \in {\mathbb {R}}^{l}$, $\mathbf {x} \in {\mathbb {R}}^{l \times n}$, in a standard linear regression, we wish to find parameters *β*_0_,***β*** such that the sum of square residuals, $ \sum _{i=1}^{l}(y_{i} - \boldsymbol {\beta _{0}} - x_{i,\cdot }^{\top } \boldsymbol {\beta })^{2}$, is minimized.

Many machine learning methods have been applied to the genetic trait prediction problem, such as Elastic-Net, Lasso, Ridge Regression [10, 11], Bayes A, Bayes B [1], Bayes *C*_*π*_ [12], and Bayesian Lasso [13, 14]. As in this work, we applied rrBLUP and SVR, we mainly focused on reviewing these two techniques.

3 $$  \begin{aligned} \underset{(\beta_{0},\boldsymbol{\beta}) \in {\mathbb{R}}^{n+1}}{min} & \quad \left[ \frac{1}{2l} \sum_{i=1}^{l} (y_{i} - \beta_{0} - x_{i,\cdot}^{\top} \boldsymbol{\beta})^{2} + \lambda P_{\alpha}(\boldsymbol{\beta})\right],\\ \end{aligned}  $$

*rrBLUP (Ridge regression BLUP)* [1, 9] is one of the most popular methods for genetic trait prediction. rrBLUP simply is ridge regression with a specific choice of *λ* in (3). Specifically, Meuwissen et al. [16] assumes that the *β* coefficients are iid from a normal distribution such that $\beta _{i} \sim N(0,\sigma _{\beta }^{2})$. Then the choice of $\lambda = {\sigma _{e}^{2}}/\sigma _{\beta }^{2}$ where ${\sigma _{e}^{2}}$ is the residual error. In this case, the ridge regression penalized estimator is equivalent to best linear unbiased predictor (BLUP) [17].

Support vector machines (SVMs) are a tool in statistics and machine learning for the task of supervised learning [18–22] used for either classification or regression. Here we are interested in the latter case. Following [23], given a training set (**x**_*i*_,*y*_*i*_), *i*=1,…*l*, where $\mathbf {x}_{i} \in {\mathbb {R}}^{n}$, the goal of *ε*-SV regression is to find a function *f*(**x**) that is at most *ε* deviation from the training data *y*_*i*_ over the training data **x**_*i*_, while remaining as flat as possible in the feature space. Training an SVM requires solving

4 $$ \begin{aligned}  \underset{\mathbf{w},b,\xi}{min} & \quad \frac{1}{2} \mathbf{w}^{\top} \mathbf{w} + C\sum_{i=1}^{l} \xi_{i} \\ \text{subject to} & \quad y_{i}(\mathbf{w}^{\top} \phi(\mathbf{x}_{i}) + b) \geq 1 - \xi_{i} - \epsilon,\\ \xi_{i} \geq 0. \end{aligned}  $$

The data vectors **x**_*i*_ are mapped to another space via the function *ϕ*, and SVM attempts to fit the data in this higher dimensional space. Thus, the choice of *ϕ*, referred to as the *kernel*, has a large impact. Four kernels are usually used:

$$\begin{array}{*{20}l} \text{Linear:} \quad & \mathbf{u}^{\top} \mathbf{v},\\ \text{Polynomial:} \quad & (\gamma \mathbf{u}^{\top} \mathbf{v} + r)^{d},\; \gamma > 0,\\ \text{Radial:} \quad & exp(-\gamma\| \mathbf{u} - \mathbf{v}\|^{2}),\; \gamma >0,\\ \text{Sigmoid:} \quad & tanh(\gamma \mathbf{u}^{\top} \mathbf{v} + r). \end{array} $$

Support vector regression involves solving Eq. 4 given training data. The vector **w**, the choice of the kernel, and the choice of kernel parameters, used previously to solve Eq. 4 gives a model capable of predicting future data.

## Encoding mechanisms and evaluation

For a linear regression problem shown in Eq. 1, different encodings would not change the regression result as the coefficients and error terms would compromise the difference of the encodings. Assuming the old encoding and new encoding for the *i*-the feature are *X*_*i*_ and *Xi*′ respectively, we could always have *Xi*′=*X*_*i*_+*Δ*_*i*_, where *Δ*_*i*_ is a vector. For example, [1, 2, 4] = [0, 1, 2] + [1, 1, 2]. Then we could have:

5 $$\begin{array}{@{}rcl@{}} Y & = & \beta_{0} + \sum \beta_{i} X_{i}' + e \\  & = & \beta_{0} + \sum \beta_{i} (X_{i} + \Delta_{i}) + e\\  & = & \beta_{0} + \sum \beta_{i} X_{i} + \sum \beta_{i} \Delta_{i} + e \\  & = & \beta_{0} + \sum \beta_{i} X_{i} + e'  \end{array} $$

where $e' = \sum \beta _{i} \Delta _{i} + e$. We can see that the regression for the new encoding and old encoding indeed differ only by the error term. As the error term *e* follows a normal distribution *N*(0,*δ*), For different encodings, the *δ* could be different, thus lead to different regression performance. Therefore encodings does matter to the regression task, as we will show in the next few sections.

### Different encoding mechanisms

In this work, we view the quantitative genetic trait prediction as a multiple regression on categorical data. Multiple regression problem on categorical data requires encoding. Various encoding mechanisms have been proposed. The most common one is called dummy encoding. In general, a categorical variable with *k* levels will be transformed into *k*−1 dichotomous variables each with two levels. For example, for a variable of three possible values, or levels, we could transform it into two dummy dichotomous variables *A* and *B*. For value one, we assign *A*=1,*B*=0. For value two, we assign *A*=0,*B*=1. For value three, we assign *A*=0,*B*=0. Thus for *n* markers, we need at least 2*n* dummy dichotomous variables. As the complexity for rrBLUP is *O*(*m*^2^), where *m* is the number of markers, the complexity with dummy encoding becomes *O*(4*n*^2^). Given in our problem setting *n* is usually tens of thousands, *O*(4*n*^2^) in reality is significantly bigger than *O*(*n*^2^). Another issue is that using dummy encoding, we are not able to obtain a single coefficient for a marker, which is generally considered as the importance of the markers for plant and animal breeding. A set of similar encoding mechanisms such as Forward Difference Coding, Backward Difference Coding, Helmert Coding, Reverse Helmert Coding, Deviation Coding, Orthogonal Polynomial Coding all have the same issues for a combination of categorical variables for similar reasons.

Another very popular encoding method that addresses the scalability issue is ordinal encoding. Ordinal encoding assumes that the categories follow certain order and then encodes the categories with numerical values such as 0, 1, 2. This is indeed the case for our problem setting where the three categories can be considered as following the order of the number of major or minor alleles. When a combination of categories are considered, each category is encoded independently. For genotype encoding, a traditional way is to encode the three categories the same way across all markers. A different encoding mechanism, Target-based encoding encodes each category as the mean of the target variable for that specific category. This encoding method allows each marker to be encoded differently. However, the order of the categories are not maintained. Thus for a combination of categorical variables, the order of the categories of each variable is relatively random.

In this work, in order to address the drawbacks of the ordinal encoding and the target-based encoding while maintaining both of their advantages, we develop two hybrid encoding methods. Assuming 0 stands for the homozygous genotype with major allele, 2 stands for the homozygous genotype with minor allele, 1 stands for the heterozygous genotype, the first hybrid method computes new encodings of genotypes at marker *i* as the follows:

6 $$\begin{array}{@{}rcl@{}} E(i, 0) & = & Ave(trait(i, 0)) \\  E(i, 2) & = & Ave(trait(i, 2)) \\  E(i, 1) & = & Ave(trait(i, \{0,1,2\}))  \end{array} $$

where *E*(*i*,0) is the new encoding for genotype of value 0 at marker *i*, *trait*(*i*,0) is the set of traits for the samples whose genotypes are 0 at marker *i*, *Ave*() is the function to compute the average value. We call this method *Hybrid One*.

The second hybrid method computes *E*(*i*,0) and *E*(*i*,2) the same as algorithm hybrid one does. However, instead of the average of the trait, *E*(*i*,1) is computed as the average of *E*(*i*,0) and *E*(*i*,2). We call this method *Hybrid Two*.

7 $$\begin{array}{@{}rcl@{}} E(i, 0) & = & Ave(trait(i, 0)) \\  E(i, 2) & = & Ave(trait(i, 2)) \\  E(i, 1) & = & \frac{E(i, 0) + E(i, 2)}{2}  \end{array} $$

We can see that for both hybrid one and hybrid two, *E*(*i*,0) and *E*(*i*,2) are computed the same as those from target-based encoding. However, target-based encoding computes *E*(*i*,1) as *Ave*(*trait*(*i*,1)) which then loses the order of the categories. For both hybrid one and hybrid two, it is guaranteed that *E*(*i*,1)=*Ave*(*trait*(*i*,{0,1,2})) and $E(i, 1) = \frac {E(i, 0) + E(i, 2)}{2}$ are in between of *E*(*i*,0) and *E*(*i*,2). Thus the order of categories is maintained. The difference is that *E*(*i*,1)=*Ave*(*trait*(*i*,{0,1,2})) is closer to *E*(*i*,0) as 0 stands for the heterozygous with major allele, where most of the samples have this genotype, thus the average of the whole trait values is close to *E*(*i*,0). On the contrary, $E(i, 1) = \frac {E(i, 0) + E(i, 2)}{2}$ requires its value as the mean of *E*(*i*,0) and *E*(*i*,2). From our experiments, the second strategy achieves slightly better performance.

**Fig. 1:**
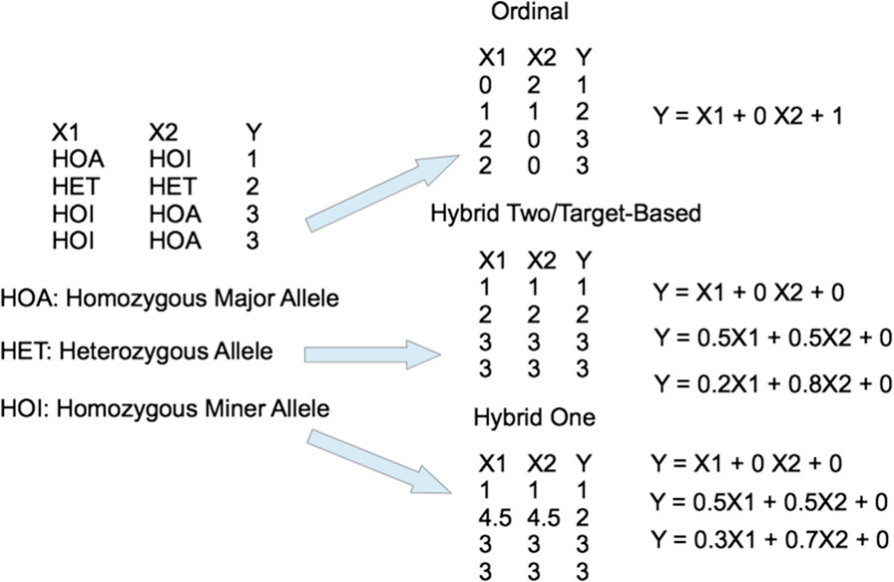
Examples of different encodings

We show an example in Fig. 1. The figure on the left shows the multiple regression problem. The figures on the right show the codings of different encoding methods. Notice we just simply give some sample regression and the regressions might not be perfect. As we can see, for ordinal encoding, *X*_1_ is positively correlated to *Y*, *X*_2_ is negatively correlated to *Y*. For the other encodings, both *X*_1_ and *X*_2_ are positively correlated to *Y*.

Notice for the ordinal encoding, we could check if the correlation between a feature and the trait is positive or not. If the correlation is negative, we could flip 0 and 2 to make the feature positively correlated to the trait. This strategy would work well for single marker model. However, it can not be extended to the epistasis model where we could have 9 values for each feature for the pairwise epistasis case and 3^*k*^ values for the *k*-way interactions.

### Performance analysis of different encoding mechanisms

As we will show later in the experiments in Section “Results”, the hybrid encoding methods are able to improve the performance not only for the epistasis model, but also for the single marker model. Next we investigate the reason that the hybrid encodings are in general superior to the other encoding methods.

#### Importance of encoding flexibility

The issue of the traditional encoding, or the ordinal encoding for multiple regression problem is that as all the categories are encoded the same way for different markers. Our encoding methods, as well as the target based encoding, encode each marker by assigning values similar to the trait values according to how the genotype categories are distributed for the marker. As we will show later in our experiments, this strategy is able to improve the correlation of the encoded features to the trait value and thus tends to improve the regression performance.

#### Importance of maintaining the order of categories

The issue of target-based encoding is that the encoded values of the categories completely depend on the trait and therefore the order of the categories are not maintained. The encoded value for the heterozygous category for target-based encoding could be smaller or greater than the values for both homozygous categories. On the contrary, for the hybrid encoding, the value for the heterozygous category is always in between of the values of the two homozygous categories and therefore the order of the categories are maintained.

To evaluate the importance of such category order maintenance, we calculate the distance between samples for different encodings, where we consider each sample as a *m* dimensional vector and *m* is the number of features. The distance is computed as the follows.

We first compute the z-score of trait value for each sample as $\frac {x-\mu }{\delta ^{2}}$, where *x* is the original trait value, *μ* is the mean of all the traits, *δ* is the standard deviation of all the traits. Then, we assign discretized values to samples according to their trait z-score using the following formula:

8 $$\begin{array}{@{}rcl@{}} discretized~value & = & \left\{ \begin{array}{l l} -1 & \quad \text{if z-score \(<\) -1}\\ 1 & \quad \text{if z-score \(>\) 1}\\ 0 & \quad \text{otherwise}\\ \end{array} \right.  \end{array} $$

Then we consider the distance of the samples with identical discretized trait value. The intuition is that when a set of samples have similar trait values and they are close to each other in the feature space, the residual error of the regression tend to be small. An extreme scenario is all the samples have the same trait value and also the same feature values, namely all these samples are identical points in the feature space, the regression will have residual error 0. On the contrary, if the samples are far from each other while they have the same trait value, the residual error tend to be large.

As for different encodings the scales of the encoded values are different, we normalize the encoded values and compute the z-score of the *j*-th marker for the *i*-th sample as $z(i, j) = \frac {(x(i, j) - \mu (j))^{2}}{var(j)}$, where *z*(*i*,*j*) is the z-score for sample *i* at marker *j*, *x*(*i*,*j*) is the encoded value of sample *i* at marker *j*, *μ*(*j*) is the mean of the encoded values for marker *j* for all samples, *var*(*j*) is the variance of the encoded values for marker *j*. Once we compute the z-score of each marker for each sample under each encoding, we measure the pairwise distance between every pair of samples *i*,*j* using Euclidean distance as $dis(i, j) = \sqrt {\sum (z(i, k) - z(j, k))^{2}}$, for 1≤*k*≤*d* and *d* is the total number of markers. Then we compute the average distance. The smaller the average distance is, the closer the samples are. As we will show later, the target-based encoding has higher sample-wise distance than the ordinal encoding and hybrid encoding, which explains the observation that the hybrid encoding methods lead to better regression performance.

As we can see, both the encoding flexibility and the order of the categories are important for multiple regression on categorical data. Our hybrid encoding methods keep the encoding flexible among markers and in the meanwhile maintain the order of the categories. Therefore our methods achieve better performance than both the ordinal encoding and the target-based encoding do.

### Extension to epistasis model

**Fig. 2:**
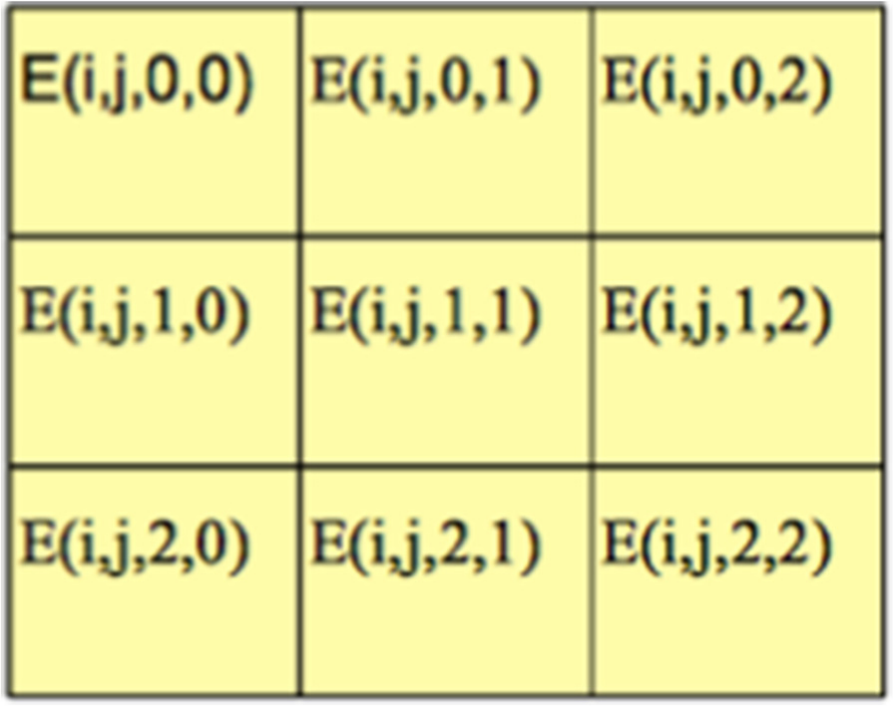
The nine possible combinations for a pair of markers *i*,*j*

The hybrid encoding strategies can be naturally extended to pairwise epistasis effects or even higher dimensional epistasis effects. As shown in Fig. 2, for pairwise epistasis effects, given the traditional encoding {0,1,2}, we have 9 possible combinations for markers *i* and *j*, organized in the 3×3 grid matrix. Assuming 0 is the traditional encoding for homozygous genotype with major allele, 2 is the traditional encoding for homozygous genotype with minor allele, 1 is traditional encoding for heterozygous genotype, then the cell (0,0) (from now on, for simplicity, we ignore the marker indices *i*,*j* for the cell) is the traditional encoding for a pair of homozygous genotypes, both with major allele, the cell (2,2) is the traditional encoding for a pair of homozygous genotypes, both with minor allele, the cell (1,2) is the traditional encoding for a pair of heterozygous genotype and homozygous genotype with minor allele. The meaning of the other cells can be inferred similarly.

Our goal is to encode each cell using the hybrid approach. We first compute the hybrid encoding for the four corner cells (0,0),(0,2),(2,0),(2,2) as the average of their corresponding trait values, as shown in Fig. 2. For example, *E*(*i*,*j*,0,0)=*Ave*(*trait*(*i*,*j*,0,0)), where *trait*(*i*,*j*,0,0) is the set of traits for the samples whose traditional genotypes at marker *i*,*j* are 0 and 0, respectively. Then for the cells (1,0),(0,1),(2,1),(1,2), we compute their hybrid encoding by extending the encoding strategy for single markers. For example, *E*(*i*,*j*,1,0)=*Ave*(*trait*(*i*,*j*,{0,1,2},0)), where *trait*(*i*,*j*,{0,1,2},0) is the set of traits for the samples whose traditional genotype at marker *i* is 0 or 1 or 2, and at marker *j* is 0, respectively. The intuition is that we consider the encoding for the three cells (0,0),(1,0),(2,0) for the marker pair *i*,*j* as fixing the genotypes for marker *j* as 0. Then the problem is converted to computing the encoding for a single marker *i*, whose genotype can be either 0, or 1, or 2. Similar encoding strategies are also applied on the cells [(0,2),(1,2),(2,2)], [(0,0),(0,1),(0,2)], [(2,0),(2,1),(2,2)] to compute the encodings for cells (1,2),(0,1),(2,1), respectively. Finally for the cell in the center (1,1), we compute its hybrid encoding as the average of all the traits, namely *E*(*i*,*j*,1,1)=*Ave*(*trait*(*i*,*j*,{0,1,2},{0,1,2})).

The above is a straight-forward extension of Hybrid One for single markers. The extension of Hybrid Two is a similar procedure with the following differences: $E(i,j,1,0) = Ave(trait(i,j,\frac {E(i,j,0,0) + E(i,j,2,0)}{2},0))$ and similar encodings for cells (1,2),(0,1),(2,1), $E(i,j,1,1) = Ave(trait(i,j,\frac {E(i,j,1,0) + E(i,j,1,2) + E(i,j,0,1) + E(i,j,2,1)}{4}))$.

The same hybrid encoding algorithm can be further extended to higher dimensional epistasis effects. In this work, we only focused on the application of the hybrid encoding algorithm on single marker and pairwise epistasis effects.

## Results

As rrBLUP is one of the most commonly used methods for genetic trait prediction, in our experiments, we evaluate the prediction accuracy for different encodings mainly using rrBLUP.

We apply the new encoding strategy to four different data sets, summarized in Table 1. We compare the performance of rrBLUP on both the traditional encoding and the two hybrid encodings and the target-based encoding. As *r*^2^, the square of the Pearson’s correlation coefficient is the most common evaluation metric for genetic trait prediction problem, we show the average *r*^2^ of 10-fold cross validation. Notice all the encodings are generated only from the training data and then applied to the test data accordingly.

**Table 1:** Summary of the data sets

Data set	Num. of markers	Num. of samples
Maize: Flint (all three datasets)	29094	261
Maize: Dent (all three datasets)	30027	261
Rice (both datasets)	36901	413
Pig (both datasets)	52842	3534
QTLMAS (both datasets)	10031	2326

The first data set is the Maize data set [7] which consists of two maize diversity panels with 300 Flint and 300 Dent lines developed for the European CornFed program. The two panels, Flint and Dent, were genotyped using a 50k SNP array, which after removing SNPs with high rate of missing markers and high average heterozygosity, yielded 29,094 and 30,027 SNPs respectively. Both of them contain 261 samples.

The second data set is the Asian rice, Oryza sativa, data set [24]. This data set was based on 44,100 SNP variants from 413 accessions of O. sativa, taken from 82 countries containing 34 phenotypes. We selected two phenotypes, one is polygenic (Protein.content), one is oligogenic (Pericarp.color). The data sets have 36,901 markers and 413 samples.

The third data set is Pig data set, which is a collection data on male and female pigs born since 2000 and was taken from [5] and consists of 3,534 animals from a single PIC nucleus pig line yielding 52,842 SNPs with five measured traits (phenotypes). Only traits 2 and 4 were selected for study here. As described in [5], genotypes were sequenced from the Illumina PorcineSNP60 chip and full pedigree information is available, which we did not use in this study. In the original study, trait 2 was rescaled by a weighted mean of corrected progeny phenotypes. Whereas trait 4 was corrected for environmental factors such as year of birth and location. Genotypes were filtered for minor allele frequency less than 0.001 and with missing genotypes less than 10 %. The original study used AlphaImpute to impute any missing data [14].

The fourth data set is QTLMAS data set, which was taken from the QTL-MAS Workshop, which was held on May 17–18, 2010 in Poznan Poland [1]. The data set consists of 3,226 individuals over five generations (F0-F4) with 20 founders, five male and 15 females. There were two phenotype traits, the first a quantitative trait and the second a binary trait. Only the first four generations (2,326 individuals) have phenotype records. The genome is approximately 500 million bp with five chromosomes, each 100 million bp. In total, each individual was genotypes for 10,031 biallelic SNPs.

For genetic prediction, to our knowledge, there is no method can achieve consistently better performance than rrBLUP does with similar running time. Also compared with rrBLUP, even for cases where the performance can be improved, most of the other methods can not make an improvement over 5 %. Thus we consider an improvement of 5 % as significant. As shown in Table 2, in general the hybrid encodings are able to improve the prediction performance and in many cases the improvement is significant. The target-based encoding is slightly better than the traditional encoding, but worse than both hybrid encodings. Thus for single marker model, the hybrid encodings are superior to the traditional encoding and the target-based encoding. The two hybrid encodings have similar performance.

**Table 2:** Performance of rrBLUP (average *r* ^2^) on the traits of four real data sets under the traditional encoding vs. the hybrid encodings vs. the target-based encoding. We also show the improvements of the hybrid encodings over the traditional encoding

Data set	Traditional encoding	Hybrid one (improvement)	Hybrid two (improvement)	Target-based
Rice: Pericarp.color	0.433	0.499 (16 %)	**0.504** (16.4 %)	0.493
Rice: Protein.content	0.176	0.176 (1 %)	**0.177** (1 %)	0.177
Pig: Trait 2	0.237	0.238 (1 %)	**0.239** (1 %)	0.236
Pig: Trait 4	0.203	**0.218** (7 %)	**0.218** (7 %)	0.207
QTLMAS: Trait 1	0.358	0.36 (1 %)	**0.361** (1 %)	0.36
QTLMAS: Trait 2	**0.187**	0.179 (-4 %)	0.18 (-4 %)	0.178
Maize: Flint 1 TASS	0.47	**0.492** (5 %)	**0.492** (5 %)	0.475
Maize: Flint 2 DMC	0.301	**0.311** (2.5 %)	0.308 (2.3 %)	0.289
Maize: Flint 3 DM_Yield	0.057	**0.07** (20 %)	0.068 (19 %)	0.062
Maize: Dent 1 Tass	0.59	0.615 (4.4 %)	**0.616** (4.4 %)	0.593
Maize: Dent 2 DMC	0.562	0.58 (3.2 %)	0.58 (3.2 %)	**0.582**
Maize: Dent 3 DM_Yield	0.321	0.343 (8.6 %)	**0.349** (8.7 %)	0.346

The bold numbers are the ones with the best performance

We also conducted SVR (support vector regression) with sigmoid kernel on all the data sets with different encodings. We show only the results for the Maize data. The results are shown in Table 3. We can see that the two hybrid encoding methods achieve almost identical accuracies, both are higher than the accuracy from the target based encoding. The traditional encoding has the worst accuracy.

**Table 3:** Performance of SVM (average *r* ^2^) on the traits of Maize data set under the traditional encoding vs. the hybrid encodings vs. the target-based encoding

Data set	Traditional encoding	Hybrid one	Hybrid two	Target-based
Maize: Flint 1 TASS	0.317	**0.344**	**0.344**	0.328
Maize: Flint 2 DMC	0.278	**0.29**	**0.29**	0.27
Maize: Flint 3 DM_Yield	0.066	**0.075**	0.074	0.05
Maize: Dent 1 Tass	0.2	**0.27**	**0.27**	**0.27**
Maize: Dent 2 DMC	0.45	0.49	0.495	**0.48**
Maize: Dent 3 DM_Yield	0.32	**0.34**	**0.34**	0.33

The bold numbers are the ones with the best performance

Next we compute the average correlation of the top-100 markers with the highest absolute correlation values (as the correlation could be either positive or negative) to the trait under different encoding methods. The results are shown in Table 4. We show only the results for the Maize data. We can see that for the ordinal encoding, the average correlations are smaller than those of the other encoding methods, indicating that by allowing encoding flexibility, we could potentially improve the regression performance. The target-based encoding has the highest average correlation. However, due to its lack of category order maintenance, its performance is worse than those from the hybrid methods. The two hybrid methods have identical average correlations which are slightly lower than that of the target-based encoding.

**Table 4:** The average correlation of the top-100 markers with the highest absolute correlation values to the trait under different encoding methods. We also show the percentage of the positively correlated features for the traditional encoding

Data set	Ordinal encoding	Hybrid one	Hybrid two	Target-based
	(positive percentage)			encoding
Maize: Flint 1 TASS	0.4	0.41	0.41	0.43
Maize: Flint 2 DMC	0.35	0.37	0.37	0.38
Maize: Flint 3 DM_Yield	0.22	0.23	0.23	0.30
Maize: Dent 1 Tass	0.36	0.37	0.37	0.38
Maize: Dent 2 DMC	0.4	0.40	0.40	0.40
Maize: Dent 3 DM_Yield	0.32	0.33	0.33	0.34

In order to show the importance of category order maintenance, we show in Table 5 the average pairwise distance of the samples for each encoding method. Due to space limit, we show only the results for the Maize data. We can see that the target-based encoding has the biggest pairwise distance while the hybrid encoding methods have lower pairwise distance. The traditional encoding has lower pairwise distance, but due to its mixture of both positively-correlated and negatively-correlated features, its performance is worse than those of the hybrid encodings.

**Table 5:** Average pairwise distance of the samples under different encoding methods

Data set	Ordinal	Hybrid one	Hybrid two	Target-based
	encoding			encoding
Maize: Flint 1 TASS	608	628	608	691
Maize: Flint 2 DMC	608	637	608	749
Maize: Flint 3 DM_Yield	608	628	608	692
Maize: Dent 1 Tass	620	657	620	1641
Maize: Dent 2 DMC	620	641	620	1725
Maize: Dent 3 DM_Yield	620	642	620	1503

We also applied the hybrid encoding strategies on the epistasis model shown in Formula 2. Due to space limit, we did not include the experimental results for the epistasis model, which will be included in the extended version of the work. However, our experiments indicate that the hybrid encoding strategies improved the prediction performance on the epistasis model as well.

## Conclusions

In this work, we showed that the quantitative genetic trait prediction problem heavily depends on the encoding of genotypes, for both single marker model and epistasis model. We developed two hybrid encoding methods which are simple but effective. Our experiments show that the hybrid encodings are able to improve the prediction accuracy for both single marker model and epistasis model. We also conducted a detailed analysis on the performance of the hybrid encodings. To our knowledge, this is the first work that discusses the effects of encodings for genetic trait prediction problem. In our future work, we would like to develop more effective encoding methods for both single marker and epistasis models. We would also like to investigate the effects of variation of allele frequency between train and test data and the effects of correlation of markers (linkage).
